# Supplementary material for: Intermediately Methylated Regions in Normal Cells Are Epimutation Hotspots in Cancer
Source: bioRxiv. 2025 Sep 23:2025.09.18.677221. Preprint. [Version 2] doi: 10.1101/2025.09.18.677221 (PMC12485763; doi:10.1101/2025.09.18.677221)
Supplement: Supplement 1 [file NIHPP2025.09.18.677221v2-supplement-1.pdf]

bioRxiv preprint doi: https://doi.org/10.1101/2025.09.18.677221; this version posted September 23, 2025. The copyright holder for this preprint (which was not certified by peer review) is the author/funder, who has granted bioRxiv a license to display the preprint in perpetuity. It is made available under aCC-BY 4.0 International license.

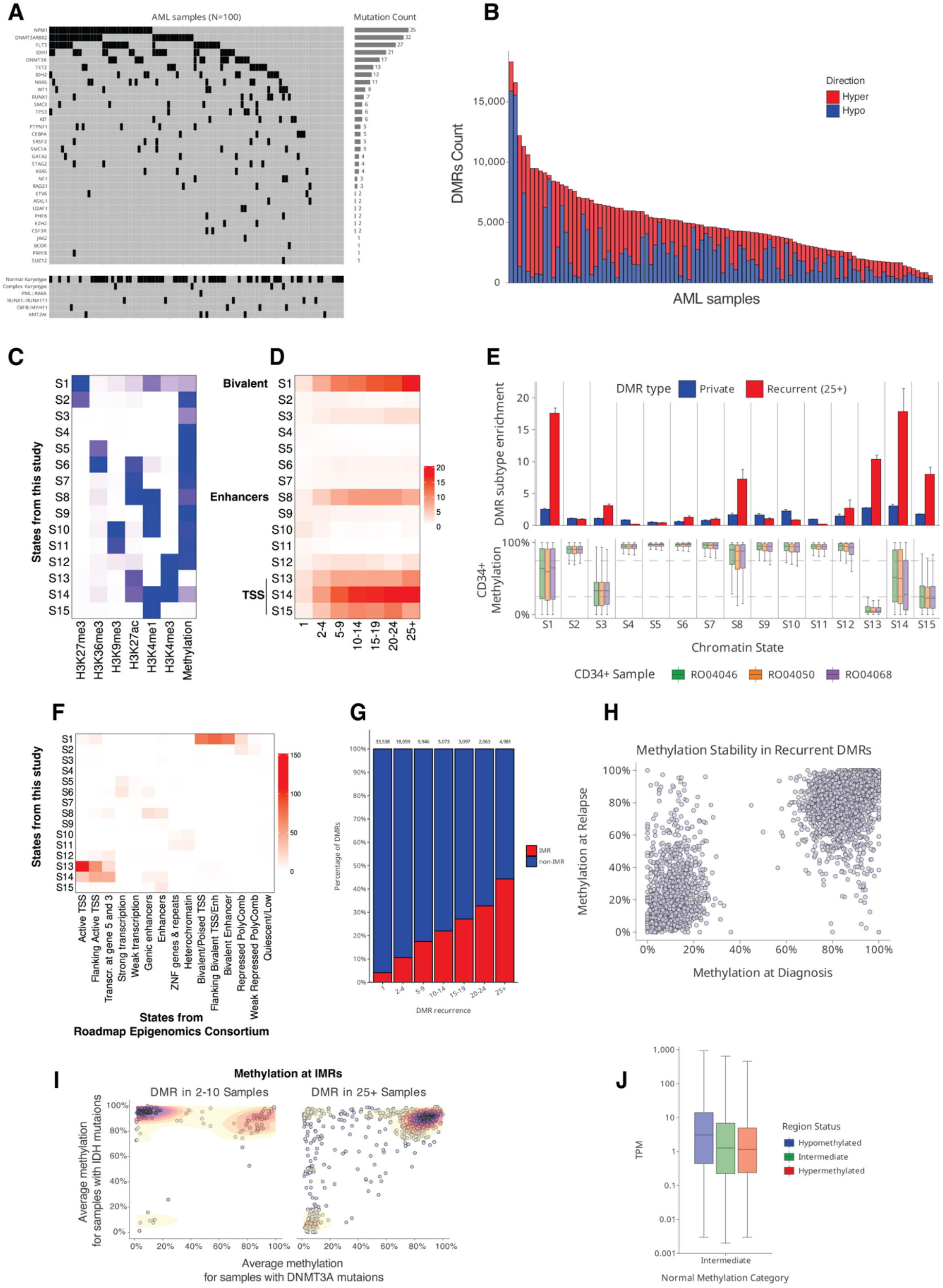

bioRxiv preprint doi: https://doi.org/10.1101/2025.09.18.677221; this version posted September 23, 2025. The copyright holder for this preprint (which was not certified by peer review) is the author/funder, who has granted bioRxiv a license to display the preprint in perpetuity. It is made available under aCC-BY 4.0 International license.

Fig. S1. Identification of differentially methylated regions in AML and their enrichment in intermediately methylated regions in normal hematopoietic cells.
(A) Mutation and karyotype status of AML samples used for WGBS analysis. (**B**) Number of DMRs identified in each AML sample compared to CD34+ cells. **(C)** Emission parameters for states S1 through S15 of a chromHMM model trained on six histone modifications (H3K4me1, H3K4me3, H3K27me3, H3K36me3, H3K9me3, and H3K27ac) and DNA methylation data from purified CD34+ HSPCs. (**D**) Heatmap of DMR enrichment grouped by recurrence frequency in chromatin states defined in HSPCs. (**E**) (Top) Relative enrichment of private and recurrent DMRs and (Bottom) Methylation levels in CD34+ (n=3) at chromatin states. For the methylation analysis, all segments within each state are included. (**F**) Heatmap showing the enrichment mapping of predefined functional genomic regions, annotated in published CD34+ data from the Roadmap Epigenomics Consortium (histone modifications only), within the chromatin states (S1-S15) defined in this study using both histone modifications and DNA methylation in CD34+ cells. (**G**) Overlap percentage of AML DMRs (grouped by recurrence frequency) with previously published intermediately methylated regions (IMRs)(*37*). (**H**) Methylation levels at IMR hotspots with altered methylation (DMRs called), identified in the presentation sample, comparing the methylation levels between presentation and relapse samples from the same patient. (**I**) Comparison of average methylation levels between samples with DNMT3A and IDH mutations, including only those with an identified differentially methylated region (DMR). The analysis distinguishes between two categories of normally intermediately methylated regions (IMRs) based on DMR recurrence: high-recurrence hotspots (DMRs in ≥25 samples) and low-recurrence regions (DMRs in 2–10 samples). Only IMRs with at least two differentially methylated samples in each mutation group were included in the comparison. Regions with low recurrence exhibit methylation patterns that appear to be mutation-driven, while highly recurrent regions are consistently hypermethylated or hypomethylated regardless of mutation type. (**J**) RNA expression levels (TPM) of genes with promoters overlapping recurrent IMR hotspots in AML samples, with samples grouped by promoter methylation status into hypomethylated (<30%), intermediately methylated (30–70%), and hypermethylated (>70%) promoter categories. $P < 10^{-8}$ for a difference in expression across these groups, based on an ANOVA.

bioRxiv preprint doi: https://doi.org/10.1101/2025.09.18.677221; this version posted September 23, 2025. The copyright holder for this preprint (which was not certified by peer review) is the author/funder, who has granted bioRxiv a license to display the preprint in perpetuity. It is made available under aCC-BY 4.0 International license.

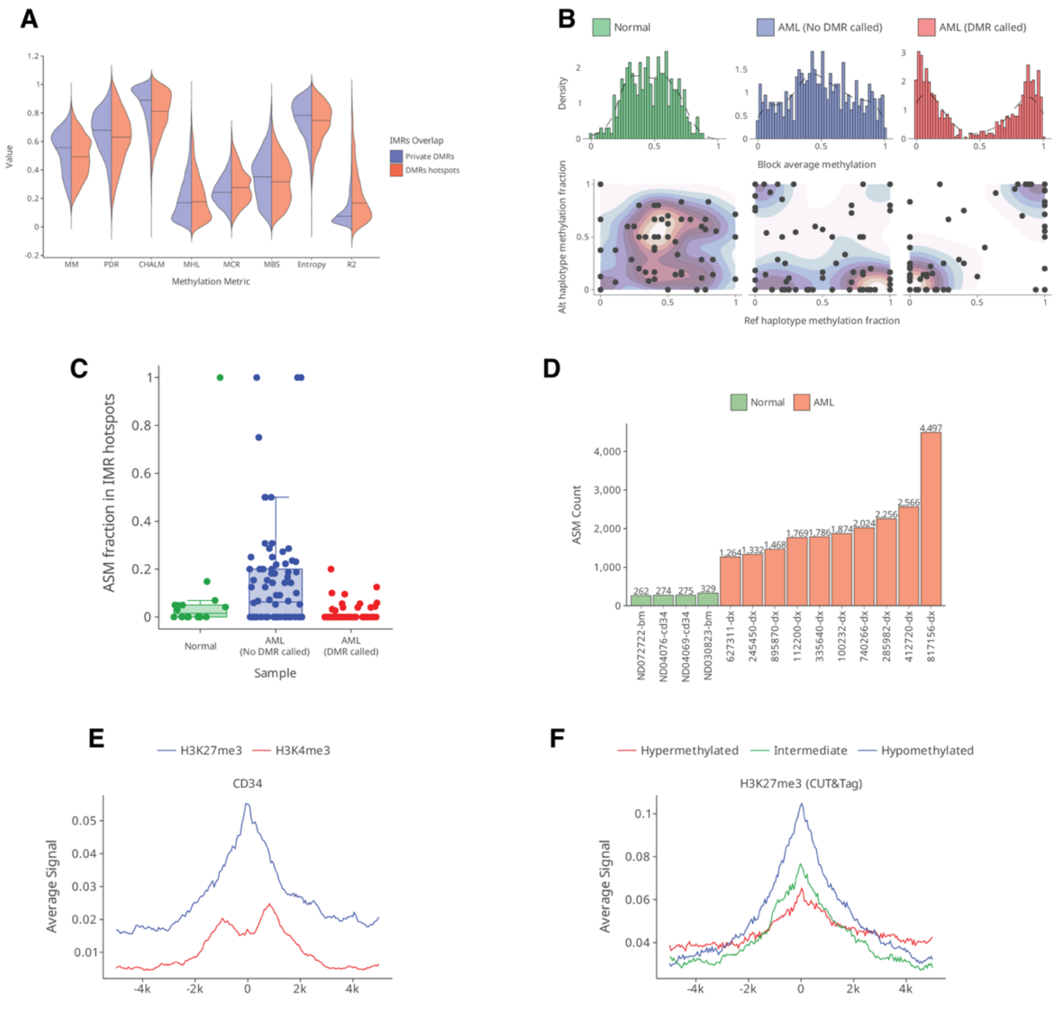

Fig. S2. Intermediately methylated regions in normal hematopoietic cells display allelic methylation patterns in AML. (A) Fragment-level WGBS methylation metrics statistics at IMRs in normal hematopoietic cells, comparing DMR hotspots vs. non-hotspot DMRs. **(B)** Methylation at IMR hotspots in normal hematopoietic cells, AML samples without differential methylation (no DMR called), and AML samples with DMRs (DMR called). Top: distribution histograms of average methylation at IMR hotspots; bottom: scatter plots comparing the average methylation of reference versus alternate haplotypes for each IMR hotspot with phased WGBS reads above the coverage threshold. **(C)** Fraction of IMR hotspots exhibiting allele-specific methylation (ASM) from WGBS in normal hematopoietic cells, AML without DMRs, and AML with DMRs. **(D)** Number of ASM regions identified from ONT haplotype-resolved methylation using the DSS R package in normal hematopoietic cells and AML samples. **(E)** Aggregated CUT&Tag signal for H3K27me3 and H3K4me3 modifications at IMR blocks in normal hematopoietic cells. **(F)** Aggregated H3K27me3 CUT&Tag signal at IMR blocks in AML samples, with blocks grouped by methylation status (hypomethylated, intermediate, or hypermethylated).

bioRxiv preprint doi: https://doi.org/10.1101/2025.09.18.677221; this version posted September 23, 2025. The copyright holder for this preprint (which was not certified by peer review) is the author/funder, who has granted bioRxiv a license to display the preprint in perpetuity. It is made available under aCC-BY 4.0 International license.

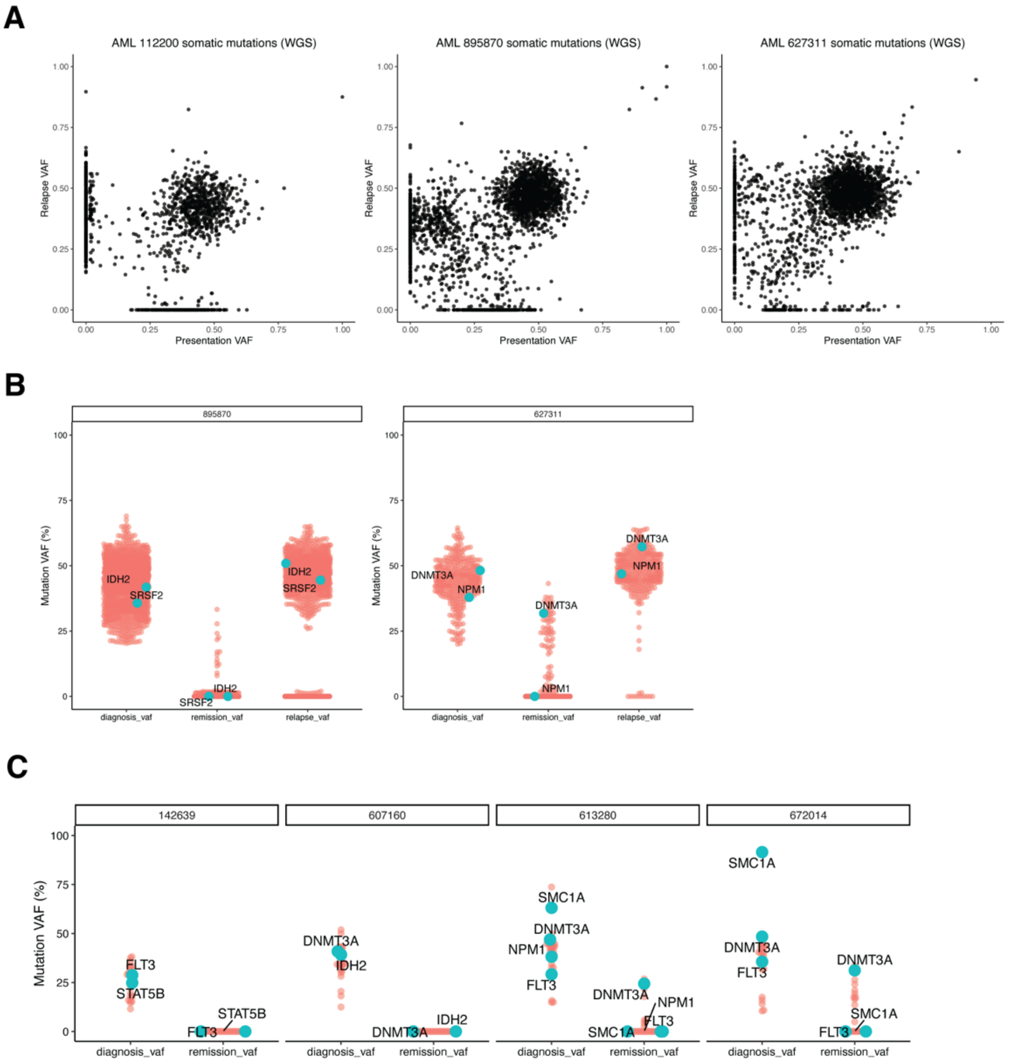

Fig. S3. IMR blocks are clonal events in AML and normal hematopoietic cell populations. **(A)** Somatic mutations from whole-genome sequencing of AML patients 112200, 895870, and 627311 at presentation and relapse. Shown are the VAFs at relapse (Y-axis) and presentation (X-axis) demonstrating their clonal relationships. **(B)** VAFs for somatic mutations from AML patient 895870 with nearly complete clearance of all variants in remission (left) vs. AML patient 627311 who had persistent variants, including *DNMT3A*^{R729W}, indicating clonal hematopoiesis at the remission timepoint. Both patients had *de novo* AML and were treated with standard “7+3” induction chemotherapy and were in complete morphologic remission at the remission time point. **(C)** Mutation clearance plots from whole-exome sequencing of 4 additional AML patients who achieved a complete morphologic remission after induction chemotherapy(*42*) .

bioRxiv preprint doi: https://doi.org/10.1101/2025.09.18.677221; this version posted September 23, 2025. The copyright holder for this preprint (which was not certified by peer review) is the author/funder, who has granted bioRxiv a license to display the preprint in perpetuity. It is made available under aCC-BY 4.0 International license.

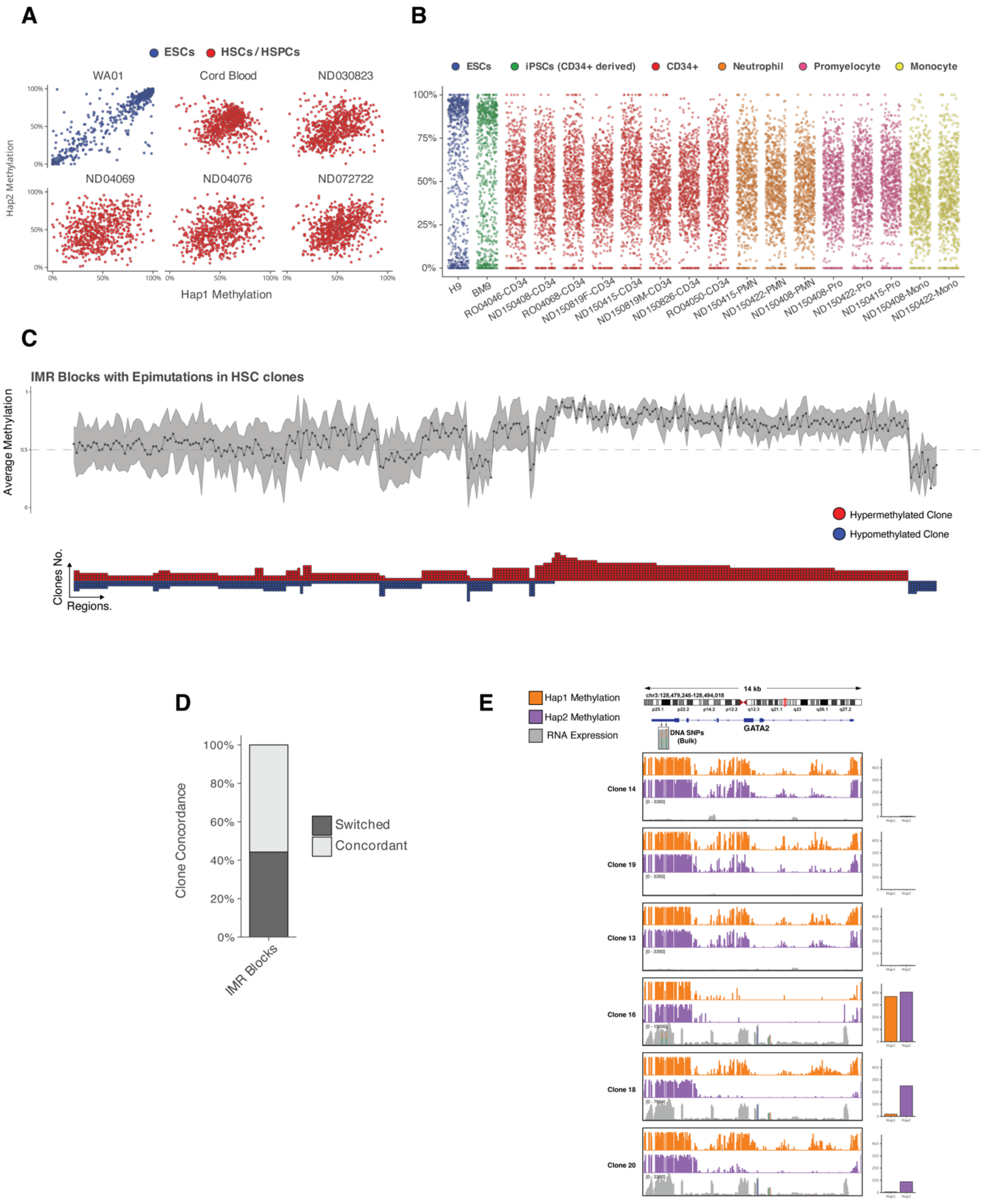

bioRxiv preprint doi: https://doi.org/10.1101/2025.09.18.677221; this version posted September 23, 2025. The copyright holder for this preprint (which was not certified by peer review) is the author/funder, who has granted bioRxiv a license to display the preprint in perpetuity. It is made available under aCC-BY 4.0 International license.

Fig. S4. Random allele-specific methylation at IMR blocks is established in single hematopoietic stem cells and is associated with allele-specific gene expression. (A) Comparison of haplotype 1 versus haplotype 2 ONT methylation at IMR blocks with intermediate methylation in bulk HSCs. Scatter plots are shown for WA01 embryonic stem cells (ESCs), cord blood HSCs, and normal donor hematopoietic stem and progenitor cells (HSPCs). **(B)** Average WGBS methylation levels at IMR blocks for H9 embryonic stem cells (ESCs), BM9 induced pluripotent stem cells (iPSCs) derived from CD34+ cells, CD34+ cells, and differentiated myeloid lineages. Each point represents the non-phased average WGBS methylation at a single single IMR for the indicated sample. **(C)** Analysis of epimutations in single HSC clones at IMR blocks with intermediate methylation. The top line plot displays the average methylation across all clones for each block. The dot plot below indicates individual clones exhibiting either hyper-methylation (red) or hypo-methylation (blue) epimutations at these blocks relative to normal HSPCs. Only IMR blocks with epimutations observed in at least four clones were included in this analysis. **(D)** Bar plot quantifying the concordance of epimutation type across clones for the IMR blocks shown in (C). “Switched” indicates regions where both hyper- and hypo-methylation events were observed in different clones, while “Concordant” indicates regions where all observed epimutations were of the same type. **(E)** Allele-specific methylation is associated with allele-specific expression at the *GATA2* locus. Tracks for representative single HSC clones show Haplotype 1 methylation (orange), Haplotype 2 methylation (purple), and RNA expression coverage (grey), with two heterozygous SNPs in the last exon used for phasing RNA expression. The bar plots on the right quantify haplotype-specific expression (TPM).

bioRxiv preprint doi: https://doi.org/10.1101/2025.09.18.677221; this version posted September 23, 2025. The copyright holder for this preprint (which was not certified by peer review) is the author/funder, who has granted bioRxiv a license to display the preprint in perpetuity. It is made available under aCC-BY 4.0 International license.

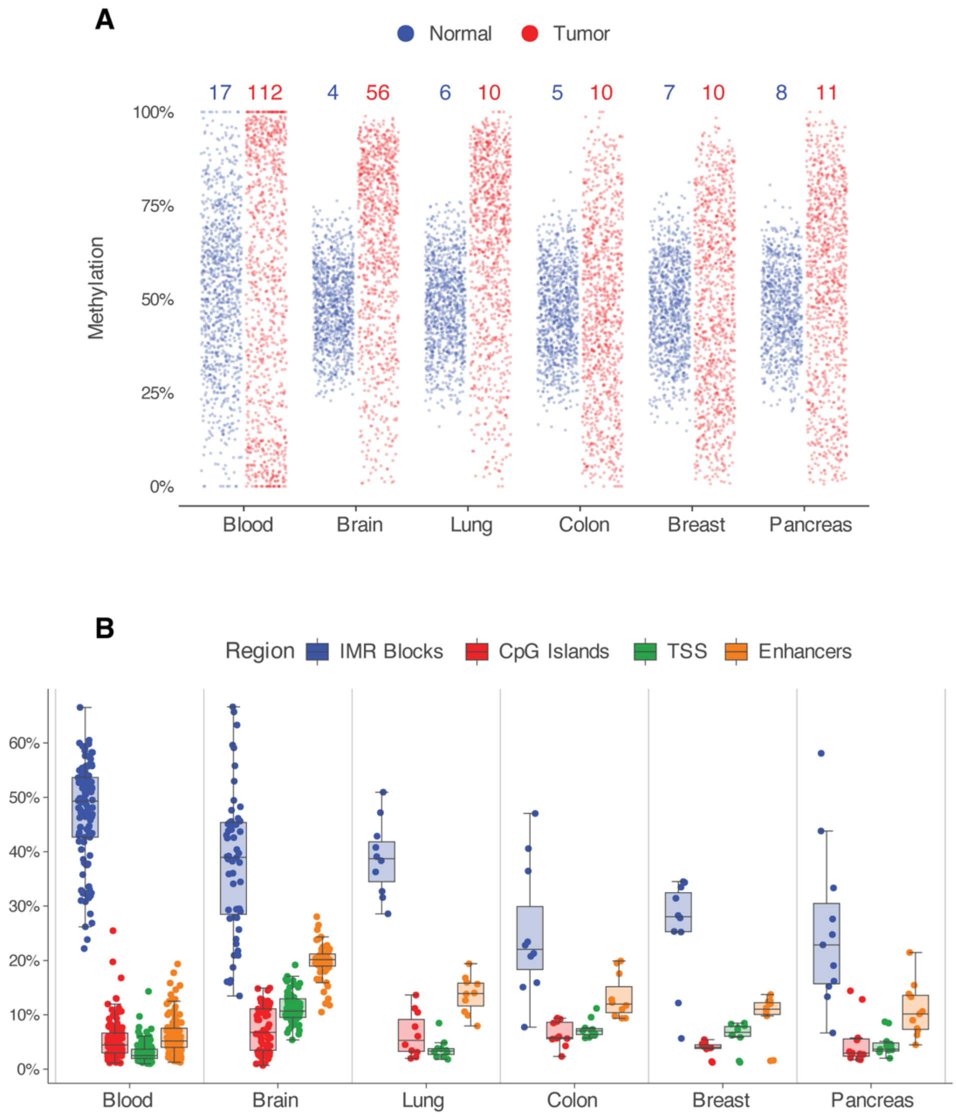

Fig. S5. Epimutations at tissue-specific IMRs using WGBS cancer datasets. (A) Distribution of average methylation levels at tissue-specific IMRs in normal tissues (blue) and their corresponding primary tumors (red), based on WGBS data. The numbers at the top of the plot indicate the number of samples included in each group. Each point represents the average methylation at a single IMR block within a sample. For visualization, 1,000 points were randomly sampled for each group. **(B)** Percentage of various genomic features exhibiting epimutations across different cancer types, based on WGBS data. The box plots compare the percentage of

bioRxiv preprint doi: https://doi.org/10.1101/2025.09.18.677221; this version posted September 23, 2025. The copyright holder for this preprint (which was not certified by peer review) is the author/funder, who has granted bioRxiv a license to display the preprint in perpetuity. It is made available under aCC-BY 4.0 International license.

epimutations within tissue-specific IMRs (IMR Blocks), CpG islands, tissue-specific transcription start sites (TSS), and tissue-specific enhancers. Each point represents an individual tumor sample.
